# Epigallocatechin-3-gallate ameliorates lipopolysaccharide-induced acute lung injury by suppression of TLR4/NF-κB signaling activation

**DOI:** 10.1590/1414-431X20198092

**Published:** 2019-06-19

**Authors:** Jia Wang, Shi Ming Fan, Jiong Zhang

**Affiliations:** 1 General Practice Center, University of Electronic Science and Technology, Sichuan Academy of Sciences & Sichuan Provincial People's Hospital, Chengdu, China; 2 Department of Respiratory Medicine, Changning Hospital of Traditional Chinese Medicine, Yibin, China; 3 Department of Nephrology, University of Electronic Science and Technology, Sichuan Academy of Sciences & Sichuan Provincial People's Hospital, Chengdu, China

**Keywords:** Epigallocatechin-3-gallate, Acute lung injury, Inflammation

## Abstract

Acute lung injury (ALI) is a serious clinical syndrome with a high rate of mortality. The activation of inflammation is well-recognized as a vital factor in the pathogenesis of lipopolysaccharide (LPS)-induced ALI. Therefore, suppression of the inflammatory response could be an ideal strategy to prevent ALI. Epigallocatechin-3-gallate (EGCG), mainly from green tea, has been shown to have an anti-inflammatory effect. The aim of the study was to explore whether EGCG alleviates inflammation in sepsis-related ALI. Male BALB/C mice were treated with EGCG (10 mg/kg) intraperitoneally (*ip*) 1 h before LPS injection (10 mg/kg, *ip*). The results showed that EGCG attenuated LPS-induced ALI as it decreased the changes in blood gases and reduced the histological lesions, wet-to-dry weight ratios, and myeloperoxidase (MPO) activity. In addition, EGCG significantly decreased the expression of pro-inflammatory cytokines tumor necrosis factor (TNF)-α, interleukin (IL)-1β, and IL-6 in the lung, serum, and bronchoalveolar lavage fluid, and alleviated the expression of TLR-4, MyD88, TRIF, and p-p65 in the lung tissue. In addition, it increased the expression of IκB-α and had no influence on the expression of p65. Collectively, these results demonstrated the protective effects of EGCG against LPS-induced ALI in mice through its anti-inflammatory effect that may be attributed to the suppression of the activation of TLR 4-dependent NF-κB signaling pathways.

## Introduction

Acute lung injury (ALI) is a common critical illness that may lead to acute respiratory failure and death. Its main pathophysiological characteristics are lung volume reduction, decreased lung compliance, and imbalance of the ventilation/blood flow ratio. Moreover, progressive hypoxemia and respiratory distress are chief clinical manifestations of ALI (1,2).

ALI is induced by a variety of clinical disorders such as infection and is characterized by widespread lung inflammation (1,2). Lipopolysaccharide (LPS), the main component of the outer membrane of Gram-negative bacteria, is an important risk factor for acute lung injury (3). Upon LPS stimulation, TLR4 is recruited and it binds to its adaptor molecules, leading to NF-κB activation and production of cytokines, such as tumor necrosis factor (TNF)-α, interleukin (IL)-1β, and IL-6. These cytokines lead to inflammation, triggering serious lung injury (4,5). Despite the significant progress in attenuating acute lung injury, the incidence and mortality of ALI in humans are still high. Therefore, it is urgent to look for a novel effective therapy for preventing acute lung injury.

Epigallocatechin-3-gallate (EGCG) is a main ingredient of green tea, a common beverage in the world (6). Several previous studies have reported that EGCG has beneficial effects in suppressing inflammation and cancer (7,8). Inflammation plays a vital role in LPS-induced ALI, whereas TLR-4/NF-κB pathway promotes inflammation in ALI (9). Increasing evidence shows that EGCG inhibits inflammation via suppressing TLR-4/NF-κB signaling (10). Therefore, abating TLR-4/NF-κB-mediated inflammation by using EGCG may be an effective way to reduce ALI. Hence, the aim of the present study was to evaluate whether EGCG decreases LPS-induced ALI in mice and whether the protective effect is associated with the attenuation of TLR-4/NF-κB pathway activation.

## Material and Methods

### Animals and experimental protocols

Thirty male BALB/C mice, 6–8 weeks old, weighing approximately 18–20 g, were purchased from Hua Fukang Experimental Animal Center, China. The mice were housed in air-filtered, temperature-controlled units with free access to food and water. The experiments were approved by the Animal Care and Use Committee of the University of Electronic Science and Technology in accordance with the Declaration of the National Institutes of Health Guide for Care and Use of Laboratory Animals.

After a minimum of 7 days of acclimation, the mice were randomly allocated to three groups with 10 mice each: 1) Control group, in which the mice were given saline (10 mg/kg, *ip*); 2) LPS group, in which the mice were given LPS (10 mg/kg dissolved in saline, *ip*); 3) EGCG group in which the rats were administered EGCG (10 mg/kg, *ip*) 1 h prior to LPS injection. The dose of EGCG was selected according to a previous study (11). At 72 h after LPS injection, the mice were anesthetized with 1% sodium pentobarbital solution (6 mL/kg). The left lung was ligated, the right lung underwent lavage three times with 1 mL of PBS, and the bronchoalveolar lavage fluid (BALF) was collected. Mice were euthanized, and blood plasma and lung tissue were collected.

### Blood gas analysis

A volume of 0.1 mL of blood was collected through the left femoral artery at 0 h (immediately after resuscitation). Anticoagulant (0.1 mL) was added immediately after each sample collection to avoid blood loss. Partial oxygen pressure (PaO_2_), partial CO_2_ pressure (PaCO_2_), pH, and respiratory rate (RR) were measured using a blood gas analyzer at the Clinical Laboratory of the Sichuan Provincial People's Hospital.

### Myeloperoxidase (MPO) activity analysis

Lung tissue was homogenized after collection. MPO activity in lung tissue was measured according to the manufacturer's instructions. The enzymatic activity was detected at 460 nm using a microplate spectrophotometer (Tecan, Switzerland).

### Wet to dry weight ratio of lung

The left lung was collected to obtain the “wet” weight. Then, the lung was dried in an oven at 120°C for 24 h and weighed to obtain the “dry” weight. Lung edema was determined by calculating the lung wet/dry (W/D) weight ratio.

### Histopathologic examination

Lung tissue was fixed overnight with 4% paraformaldehyde, dehydrated in ascending grade alcohol, embedded in paraffin, and cut into 5-μm sections. The sections were stained with hematoxylin and eosin (Solarbio, China). Pathological changes of the lung tissues were observed by a pathologist via a light microscope (Olympus, Japan). The severity of microscopic injury was graded from 0 (normal) to 4 (severe) in the following categories: interstitial edema, hemorrhage, hyaline membrane formation, necrosis, and congestion. The total score was calculated by adding the individual scores of each category (12).

### Collection of lung tissue protein and western blot analysis

Lung tissue samples were harvested at 72 h after LPS injection and frozen in liquid nitrogen immediately until later homogenization. To extract the total protein from lung tissue, protein concentrations were determined by BCA protein assay kit (Bi Yun-Tian, Shang Hai, China) and equal amounts of protein were loaded per well on a 10% sodium dodecyl sulphate polyacrylamide gel (SDS-PAGE). Subsequently, proteins were transferred onto nitrocellulose membranes. The membranes were blocked with 5% non-fat dried milk and 1% BSA. The membranes were washed in Tris-buffered saline with Tween 20 and incubated in 5% skim milk (Sigma, USA) at room temperature for 2 h on a rotary shaker, followed by TBST washing three times. Incubations with polyclonal antibodies specific for TLR-4 (1:500), MyD88 (1:500), TRIF (1:500), p-p65 (1:1000), p65 (1:1000), IκB-α, and β-actin (1:1500) in diluent buffer (5% skim milk in TBST) were performed overnight at 4°C. Then, the membrane was washed with TBST followed by incubation with the peroxidase-conjugated secondary antibody at room temperature for 1 h. β-actin (1:3000, Abmart, China) was used for normalization. The reactive bands were visualized using the ECL-Plus reagent (Amersham, USA) as instructed. The density of each reactive band was quantified using the Labworks image acquisition platform and its related analytic software (UVP, USA)

### ELISA analysis

Levels of inflammatory mediators (TNF-α, IL-6, and IL-1β) in the serum and BALF were quantified using specific ELISA kits for rats according to the manufacturer's instructions (Biosource International Inc., USA).

### Real-time PCR analysis

Total RNA was isolated from renal tissues using Trizol according to the manufacturer's instructions (Takara, Japan). Four micrograms of total RNA were reverse transcribed into cDNA using the PrimeScript RT Master Mix (Takara) as instructed. Real-time PCR amplifications were carried out using the ABI 7500 system (Applied Biosystems, USA). PCR primers (Invitrogen, USA) for all analyzed genes are shown in Table 1. PCR was conducted at 95°C for 30 s, followed by 40 cycles at 95°C for 5 s, 60°C for 34 s, and 95°C for 15 s. The amount of mRNA for each gene was normalized by β-actin, and the relative expression levels were calculated using the 2^−ΔΔCt^ method (13).

**Table 1.** Primers used for real-time PCR analysis.

Gene	Sense strand sequence	Anti-sense strand sequence
TNF-α	CTGAACTTCGGGGTGATCGG	GGCTTGTCACTCGAATTTTGAGA
Il-1β	AGCTTCCTTGTGCAAGTGTCT	GACAGCCCAGGTCAAAGGTT
IL-6	CTGCAAGAGACTTCCATCCAG	AGTGGTATAGACAGGTCTGTTGG
β-actin	AGAGGGAAATCGTGCGTGAC	CAATAGTGATGACCTGGCCGT

### Statistical analysis

Data were analyzed by GraphPad Prism (version 5.0, USA) and are reported as means±SE. Statistical significance was analyzed using one-way analysis of variance (ANOVA) followed by the Dunnett's test. P<0.05 was considered statistically significant.

## Results

### Effect of EGCG on blood gases

Blood gas analysis is an important strategy to evaluate lung function (11). Compared with the Control group, the LPS group had higher levels of PaCO_2_ and RR, and lower levels of PaO_2_ and pH. However, compared with the LPS group, the EGCG group had lower levels of PaCO_2_ and RR, with higher levels of PaO_2_ and pH as shown in Table 2. This indicated that EGCG can improve LPS-induced obstruction of respiratory function and acid-base imbalance.

**Table 2.** Comparison of blood gas analysis of the three groups.

Group	PaO_2_ (mmHg)	PaCO_2_ (mmHg)	pH	RR
Control	110±5.2	32±2.8	7.37±0.02	20±0.3
LPS	65±4.3*	47±5.1*	6.16±0.05*	24±1.2*
EGCG	90±4.8^#^	40±3.1^#^	7.36±0.04^#^	20±0.4^#^

Data are reported as means±SE. Control group: mice were given saline; LPS group: mice were given lipopolysaccharide (LPS); EGCG group: mice were given epigallocatechin-3-gallate. *P<0.05, LPS *vs* Control; ^#^P<0.05, EGCG *vs* LPS (ANOVA). PaO_2_: partial oxygen pressure; PaCO_2_: partial CO_2_ pressure; RR: respiratory rate.

### Effect of EGCG on histopathologic changes

**Figure 1.**
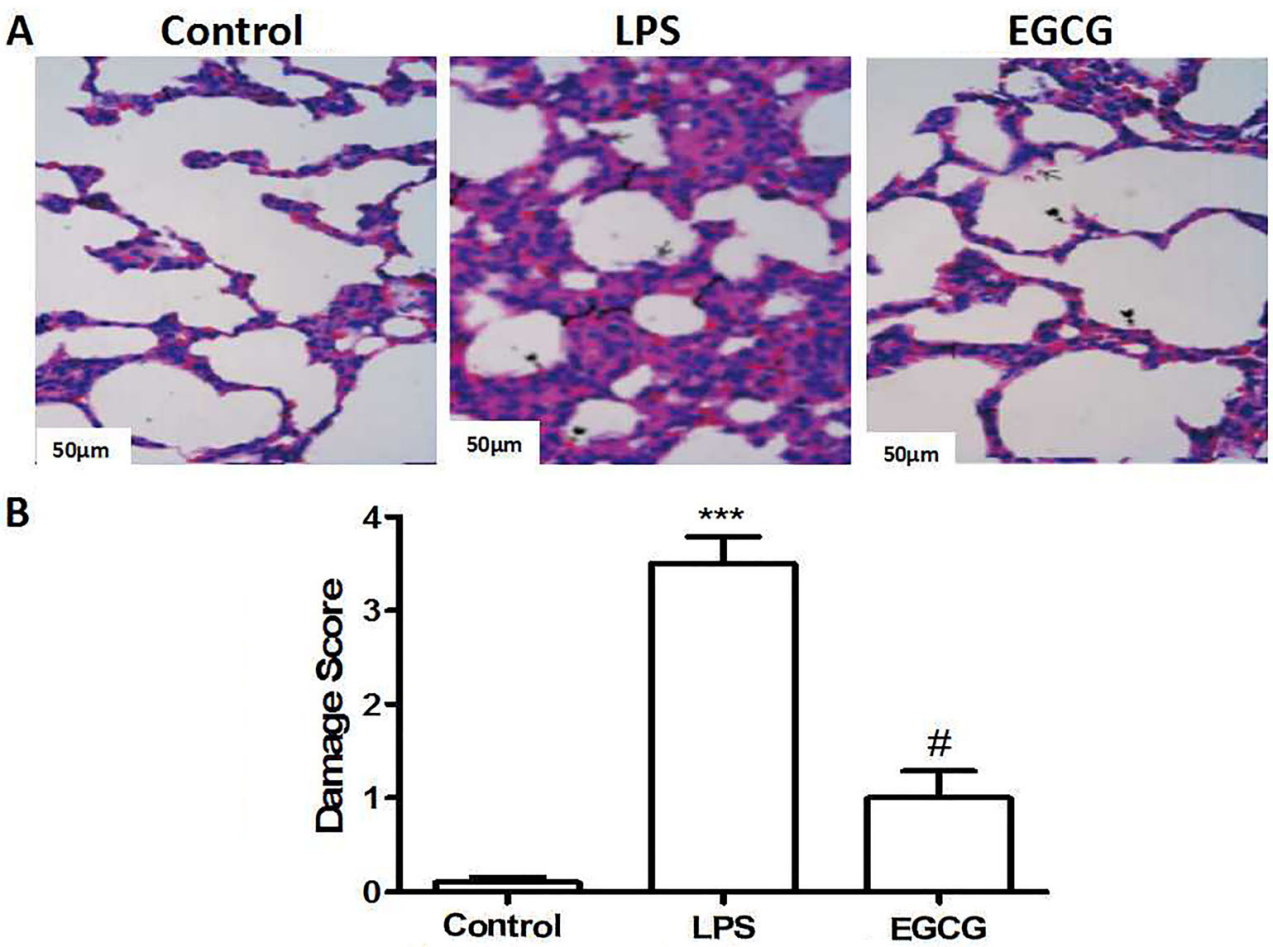
Effect of epigallocatechin-3-gallate (EGCG) pretreatment on lipopolysaccharide (LPS)-induced acute lung injury determined by histological damage score. **A**, Representative microphotographs using hematoxylin and eosin staining taken 72 h after LPS injection (bar: 50 μm). **B,** Semi-quantitative assessment of the histological lesions based on tubular necrosis. Data are reported as means±SE (n=10). ***P<0.001, compared to Control; ^#^P<0.05, compared to LPS (ANOVA).

Compared with the Control group, lung specimens from the LPS group displayed significant histopathological changes that included increased infiltration of inflammatory cells into the alveolar and interstitial spaces, severe hemorrhage in the alveolus collapse, alveolus and interstitial edema, and widespread increased alveolar wall thickness. As for the EGCG-treated groups, the severity of histopathologic changes in lung tissues was less than that in the LPS group, especially for inflammatory cell infiltration and hemorrhage (Figure 1A). Semi-quantitative assessment of histological lesions showed a significantly higher score in the LPS-treated mice compared to the EGCG-treated mice and Control mice (Figure 1B).

### Effect of EGCG on pulmonary edema and lung MPO activity

As shown in Table 3, after LPS injection, the lung wet-to-dry weight ratios and MPO activity of mice were significantly increased. However, the ratios and MPO activity were significantly decreased by EGCG pretreatment.

**Table 3.** Comparison of wet/dry (W/D) weight ratio and myeloperoxidase (MPO) activity of the three groups.

Group	W/D	MPO activity
Control	4.25±0.08	0.25±0.05
LPS	5.13±0.09*	1.12±0.07***
EGCG	4.39±0.07^#^	0.45±0.06^##^

Data are reported as means±SE. Control group: mice were given saline; LPS group: mice were given lipopolysaccharide (LPS); EGCG group: mice were given epigallocatechin-3-gallate. *P<0.05, ***P<0.001 LPS *vs* Control; ^#^P<0.05, ^##^P<0.01 EGCG *vs* LPS (ANOVA).

### Effect of EGCG on the expression of inflammation markers

Compared with the Control group, the LPS group displayed higher levels of TNF-α, IL-1β, and IL-6 in the lung, serum, and BALF. However, these expressions were significantly reduced by EGCG pretreatment (Tables 4–6).

**Table 4.** Relative expression inflammatory cytokines in the lung of mice determined by RT-PCR.

Group	TNF-α (mRNA)	IL-6 (mRNA)	IL-1β (mRNA)
Control	1.02±0.12	1.00±0.15	1.05±0.05
LPS	13.50±1.00***	8.50±0.55***	4.90±0.61***
EGCG	5.50±0.55^#^	5.00±0.25^#^	2.40±0.35^#^

Data are reported as means±SE. Control group: mice were given saline; LPS group: mice were given lipopolysaccharide (LPS); EGCG group: mice were given epigallocatechin-3-gallate. ***P<0.001 LPS *vs* Control; ^#^P<0.05 EGCG *vs* LPS (ANOVA). TNF-α: tumor necrosis factor-α; IL: interleukin.

**Table 5. t05:** Expression of tumor necrosis factor-α (TNF-α), interleukin (IL)-1β, and IL-6 in serum of mice determined by ELISA.

Group	TNF-α (pg/mL)	IL-6 (pg/mL)	IL-1β (pg/m/L)
Control	115±10	100±15	105±10
LPS	1300±250***	450±50***	850±100***
EGCG	650±55^#^	260±30^#^	550±50^#^

Data are reported as means±SE. Control group: mice were given saline; LPS group: mice were given lipopolysaccharide (LPS); EGCG group: mice were given epigallocatechin-3-gallate. ***P<0.001 LPS *vs* Control; ^#^P<0.05 EGCG *vs* LPS (ANOVA).

**Table 6.** Relative expression of tumor necrosis factor-α (TNF-α), interleukin (IL)-1β, and IL-6 in the bronchoalveolar lavage fluid determined by ELISA.

Group	TNF-α (pg/mL)	IL-6 (pg/mL)	IL-1β (pg/mL)
Control	1.25±0.24	1.23±0.11	1.03±0.12
LPS	8.56±1.34***	3.86±0.49***	4.75±0.45***
EGCG	4.28±0.51^#^	1.85±0.22^#^	2.16±0.31^#^

Data are reported as means±SE. Control group: mice were given saline; LPS group: mice were given lipopolysaccharide (LPS); EGCG group: mice were given epigallocatechin-3-gallate. ***P<0.001 LPS *vs* Control; ^#^P<0.05 EGCG *vs* LPS (ANOVA).

### Effect of EGCG on LPS-induced activation of TLR-4/NF-κB signaling

**Figure 2.**
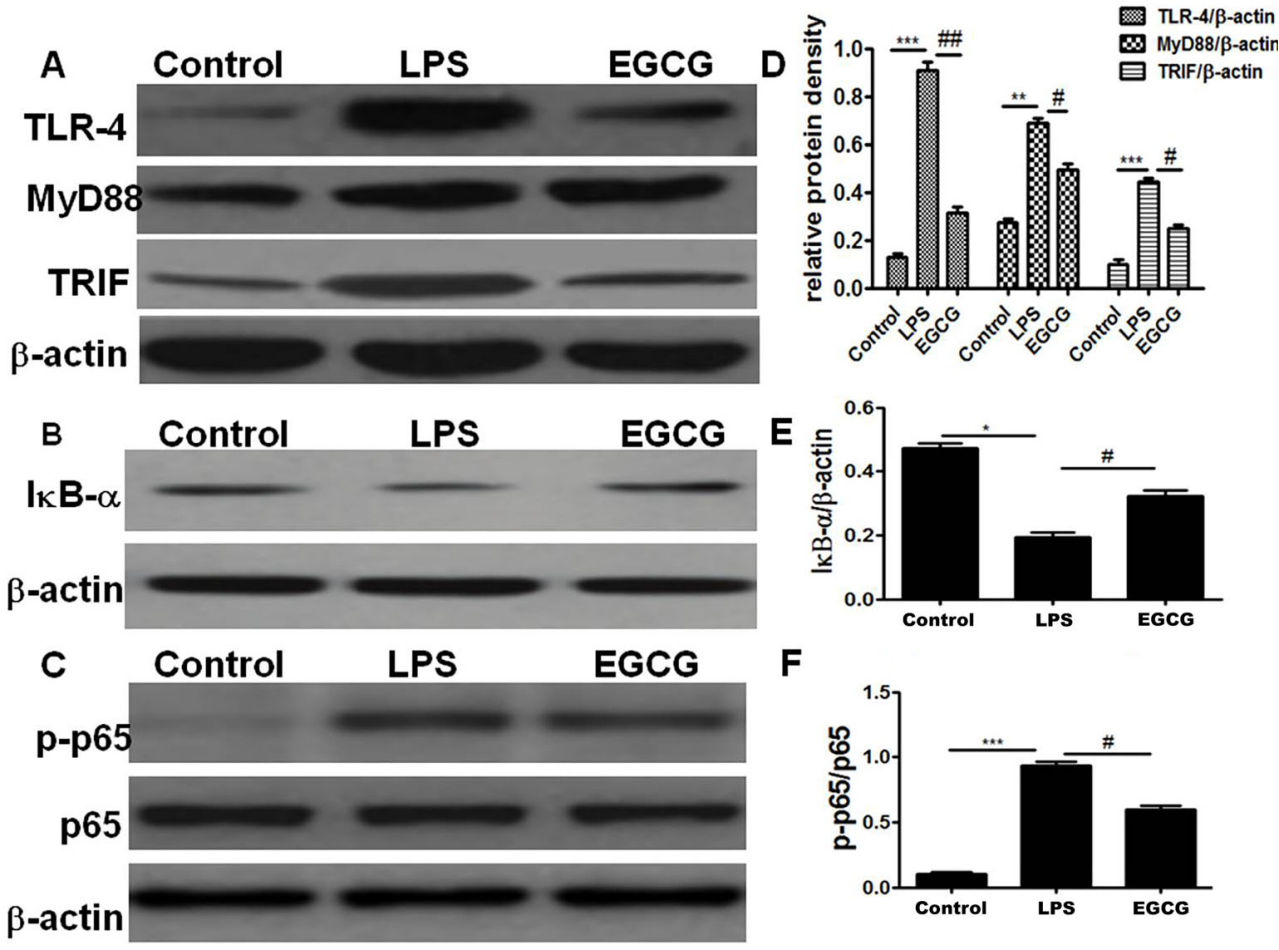
Effect of epigallocatechin-3-gallate (EGCG) pretreatment on TLR-4/NF-κB signaling after acute lung injury induced by lipopolysaccharide (LPS). **A** to **C:** Western blot analysis was employed for expression of TLR4, MyD88, TRIF, IκB-α, p-p65, and p65, normalized by β-actin. **D** to **F:** Semi-quantitative analysis of 10 animals studied in each group for each protein. Data are reported as means±SE. ***P<0.001, **P<0.01, *P<0.05 compared to Control; ^##^P<0.01, ^#^P<0.05 compared to LPS (ANOVA).

Compared with the Control group, the LPS group displayed higher levels of TLR-4, MyD88, TRIF, and p-p65, and lower expression of IκB-α. EGCG pretreatment suppressed the effect of LPS in the mice as manifested by lower expression levels of TLR-4, MyD88, TRIF, and p-p65, and higher expression of IκB-α in the EGCG group than the LPS group. Moreover, there was no difference in the expression of p65 among the three groups (Figure 2A to F). The results implied that EGCG pretreatment inhibited LPS-induced activation of TLR-4/NF-κB signaling.

## Discussion

ALI is a critical respiratory disease and a frequent complication following sepsis, which is caused mainly by LPS (1). Previous studies have shown that ALI induced by LPS is associated with increased inflammatory cell infiltration and marked lung injury that is characterized by the changes in histological and blood gas markers (1,2). Our results indicated that EGCG prevents LPS-induced ALI in our model.

LPS released by Gram-negative bacilli is the main cause of ALI (11). After entering the body, LPS is recognized by pattern recognition receptors (PRRs), which can carry out inflammation signal transduction, leading to the secretion of TNF-α, IL-1β, IL-6, and other pro-inflammatory transmitters. The imbalance of pro-inflammatory and anti-inflammatory transmitters in the body is key in the development of ALI (4,5). TNF-a can stimulate macrophages and endothelial cells to secrete large amounts of pro-inflammatory cytokines such as IL-1β, IL-8, and secondary inflammatory transmitters such as platelet activating factor, prostaglandin, and nitric oxide (14). IL-1β can enhance the sensitivity of tissue cells to TNF-α and stimulate other inflammatory transmitters such as TNF-α, IL-8, E-selectin, and P-selectin. IL-6 can induce acute inflammatory response, promote the activation and aggregation of neutrophils, cause pulmonary edema, and lead to systemic inflammatory response (4,14). These three factors promote each other and initiate the inflammatory cascade reaction leading to ALI.

TLR4/NF-kB pathway is a classical pathway that initiates intracellular inflammatory signal transduction and plays an important role in LPS-induced ALI (15,16). TLR4 has been identified as the most important PRR that recognizes LPS and mediates inflammation signal transduction. After the combination of TLR4 and LPS, the main results are as follows: mycloid differentiation factor 88 (MyD88) pathway transports signals to TNF receptor-associated factor-6 (TRAF6) step-by-step. Activated TRAF6, TAK1, and TAK1 binding protein 1, 2, and 3 (TAK1-binding protein TAB) form a compound with phosphorylated IκB, leading to degradation of IκB and activation of NF-κB (15,16). Normally, the term NF-κB refers to the p50/p65 heterodimer with major biological activity in the members of the NF-κ B/Rel family (17). Activated NF-κB translocates rapidly into the nucleus and increases the expression of pro-inflammatory cytokines such as IL-1β, TNF-α, IL-6, and IL-1β. (17). The MPO level can be used to evaluate the activation of polymorphonuclear leukocytes. An essential feature of LPS-induced ALI is the secretion of inflammatory mediators including IL-6, IL-1β, and TNF-α by the TLR4/MyD88 signal pathway, which could enlarge the inflammatory cascade and boost up neutrophil transfer into the alveoli and impair the lung (15,16).

The result of our study showed that EGCG pretreatment can suppress inflammation in LPS-induced ALI by suppressing the activation of TLR4/MyD88/NF-κB signal pathway.

A previous study demonstrated that inhibition of TLR-4/NF-KB signaling can attenuate LPS-induced ALI, and EGCG precondition can reduce LPS-induced ALI in a murine model at the dose of 10 mg/kg (18). Therefore, we did not perform a study to demonstrate that suppressing the TLR-4/NF-KB signal pathway can decrease LPS-induced inflammatory response in ALI and explore the best dose of EGCG on the LPS-induced ALI in a mouse model. EGCG could regulate additional pathways other than TLR-4/NF-KB signaling to alleviate inflammation (19). Moreover, the study only demonstrated that EGCG precondition can prevent LPS-induced ALI. However, it is uncertain whether EGCG can treat the condition. Therefore, further studies need to focus on the pathways associated with suppressing inflammation with EGCG, the best dose of EGCG precondition, and the treatment effect of EGCG on LPS-induced ALI in a mouse model.

In summary, we demonstrated that pretreatment with EGCG attenuated ALI induced by LPS as manifested by less pathological and pulmonary edema, changes. The protective mechanism was associated with suppressing TLR-4/NF-κB-p65 pathway mediating inflammation. Our study indicated that EGCG may be an effective therapy for reducing ALI in clinical practice.
